# Delivering Brief Cognitive Behavioral Therapy (CBT‐T) for Eating Disorders: Examining Real‐World Outcomes of a Large‐Scale Training Program

**DOI:** 10.1002/eat.24498

**Published:** 2025-07-02

**Authors:** Laura Dixon, Colby Price, Sara Bartel, Anastasia Harris, Marika Schenkels, Toni Spinella, Abraham Nunes, Sarrah I. Ali, Glenn Waller, Jessica Wournell, Susan Gamberg, Aaron Keshen

**Affiliations:** ^1^ Nova Scotia Provincial Eating Disorder Service Nova Scotia Health Halifax Canada; ^2^ Department of Psychiatry Dalhousie University Halifax Nova Scotia Canada; ^3^ Department of Psychology Florida State University Tallahassee Florida USA; ^4^ School of Psychology University of Sheffield Sheffield UK

**Keywords:** accessibility, CBT‐T, cognitive behavioral therapy‐ten, effectiveness, implementation, treatment outcomes

## Abstract

**Objective:**

Cognitive Behavioral Therapy‐Ten (CBT‐T) is a 10‐session manualized eating disorder (ED) treatment protocol for nonunderweight EDs. CBT‐T was developed to increase access to treatment and reduce wait times, as it can be delivered in half the time as existing CBT approaches for EDs. To improve access to treatment, the Nova Scotia Eating Disorder Provincial Service trained 36 clinicians through a 10‐month CBT‐T training program and offered CBT‐T provincially. This study examines changes in ED psychopathology, binge eating, compensatory behaviors, anxiety, and depression in a transdiagnostic cohort of adult patients treated with CBT‐T. Further, an exploratory analysis of predictors of treatment outcome was conducted.

**Methods:**

A retrospective chart review was conducted on adults who began CBT‐T between July 2022 and March 2024. Participants completed routine outcome measures per the CBT‐T manual. Mixed‐effects models examined symptom changes over time, along with predictors of treatment outcome, dropout, and extension.

**Results:**

A total of 267 patients started CBT‐T. Significant reductions in ED psychopathology, binge eating, and compensatory behaviors, anxiety, and depression were observed throughout treatment. Effect sizes were large to very large at the end of treatment for primary and secondary outcomes. Early change in ED psychopathology predicted better outcomes, whereas diagnoses of anorexia nervosa and atypical anorexia nervosa were associated with higher dropout rates.

**Discussion:**

Findings support that CBT‐T may be an effective, scalable treatment associated with significant symptom reductions corresponding to large effect sizes. Future research should explore adaptations to improve retention, especially for those with anorexia nervosa and atypical anorexia nervosa.

**Summary**

- This study demonstrates that CBT‐T in a real‐world clinical setting is associated with significant reductions in eating disorder psychopathology, binge eating, and compensatory behaviors, anxiety, and depression.
- Clinicians were trained through a large‐scale CBT‐T training program, which supports the feasibility of widespread implementation of CBT‐T in a publicly funded healthcare setting.
- Findings indicate that early improvement in ED psychopathology is the strongest predictor of positive outcomes, reinforcing the importance of early engagement in therapy.
- Higher dropout rates among individuals with anorexia nervosa and atypical anorexia nervosa highlight the need for targeted engagement strategies for these populations.

## 1 Introduction

The point prevalence of eating disorders (EDs) in North America is 4.6%, resulting in high demand for ED services (Galmiche et al. 2019). Effective outpatient treatments that can be widely and efficiently distributed are needed to improve access to care, particularly in publicly funded healthcare systems with limited resources. Currently, cognitive behavioral therapy for eating disorders (CBT‐ED) is the recommended psychotherapy for those with nonunderweight EDs and one potential approach for underweight EDs (Crone et al. 2023; National Institute for Health and Care Excellence 2017). CBT‐ED encompasses evidence‐based protocols such as enhanced CBT developed by Fairburn (CBT‐E; 2008) and CBT for EDs developed by Waller (2007). Reviews and meta‐analyses continue to demonstrate CBT‐ED effectively improves ED symptoms Öst et al. (2024).

The recommended length of CBT‐ED for nonunderweight EDs is 20 sessions and research suggests clinicians routinely extend CBT‐ED beyond 20 sessions (Cowdrey and Waller 2015). Therapists may extend treatment beyond recommended durations with the aim of improving patient outcomes, particularly if progress has been limited. However, there is little evidence to suggest longer treatments improve outcomes (Bell et al. 2017). Consequently, CBT‐ED is often expensive to deliver, and its length can contribute to long wait times (Waller et al. 2019).

To address these barriers, a 10‐session version of CBT‐ED called CBT‐Ten (CBT‐T) was developed (Waller et al. 2019). CBT‐T is a manualized outpatient treatment for nonunderweight EDs that includes foundational treatment components of CBT‐ED with an added focus on early change, exposure, and inhibitory learning principles. CBT‐T can be delivered by a range of healthcare providers, including those who are not experts in EDs (Waller et al. 2019).

A systematic review and meta‐analysis by Keegan et al. (2022) examined outcomes from 10 available studies of CBT‐T (*N* = 565). To complement this, a narrative synthesis by Paphiti and Newman (2023) evaluated 555 individuals across eight studies. Both reviews concluded that CBT‐T is effective for a range of nonunderweight EDs, including bulimia nervosa (BN), binge eating disorder (BED), and other specified feeding or eating disorders (OSFED). Key treatment targets across studies included eating disorder psychopathology, eating disorder behaviors (i.e., binge eating/purging), psychosocial impairment, depression, and anxiety. Keegan et al. (2022) reported medium to very large pooled effect sizes for these outcomes, with improvements maintained at follow‐up. For example, pooled Hedges's *g* was −1.49 for eating disorder psychopathology, −1.20 for reductions in objective binge eating frequency, and −0.78 for vomiting frequency. Furthermore, CBT‐T produces results comparable to CBT‐ED (Tatham et al. 2020). CBT‐T is also acceptable and effective for adolescents and young adults with EDs (Allen et al. 2024).

This present study describes a retrospective chart review of adults treated with CBT‐T in the Nova Scotia Eating Disorder Provincial Service (NSEDPS) between July 2022 and March 2024. The objectives of this study are to (1) examine the outcomes from CBT‐T delivered by clinicians trained in a CBT‐T training program and (2) to better understand the predictors of treatment outcome.

## 2 Methods

### 2.1 Ethical Considerations

This retrospective chart review received approval from the Nova Scotia Health Research Ethics Board.

### 2.2 Participants and Procedures

Eligible participants included NSEDPS patients aged 18 and older who began CBT‐T and completed online routine outcome measures between July 1, 2022, and March 30, 2024. All patients met criteria for Diagnostic and Statistical Manual of Mental Disorders, Fifth Edition (DSM‐5; American Psychiatric Association 2013) EDs based on clinical interview. The NSEDPS primarily offers individuals with anorexia nervosa (AN) inpatient or day treatment as a first‐line treatment option. Patients with BMI greater than 17.5 kg/m^2^ were offered inpatient, day treatment, or CBT‐T. In 2023, our service transitioned to implementing CBT‐AR (Thomas and Eddy 2019) as standard treatment for individuals with avoidant/restrictive food intake disorder (ARFID). Prior to this, individuals with ARFID were offered CBT‐T and are therefore included in this sample.

**FIGURE 1:**
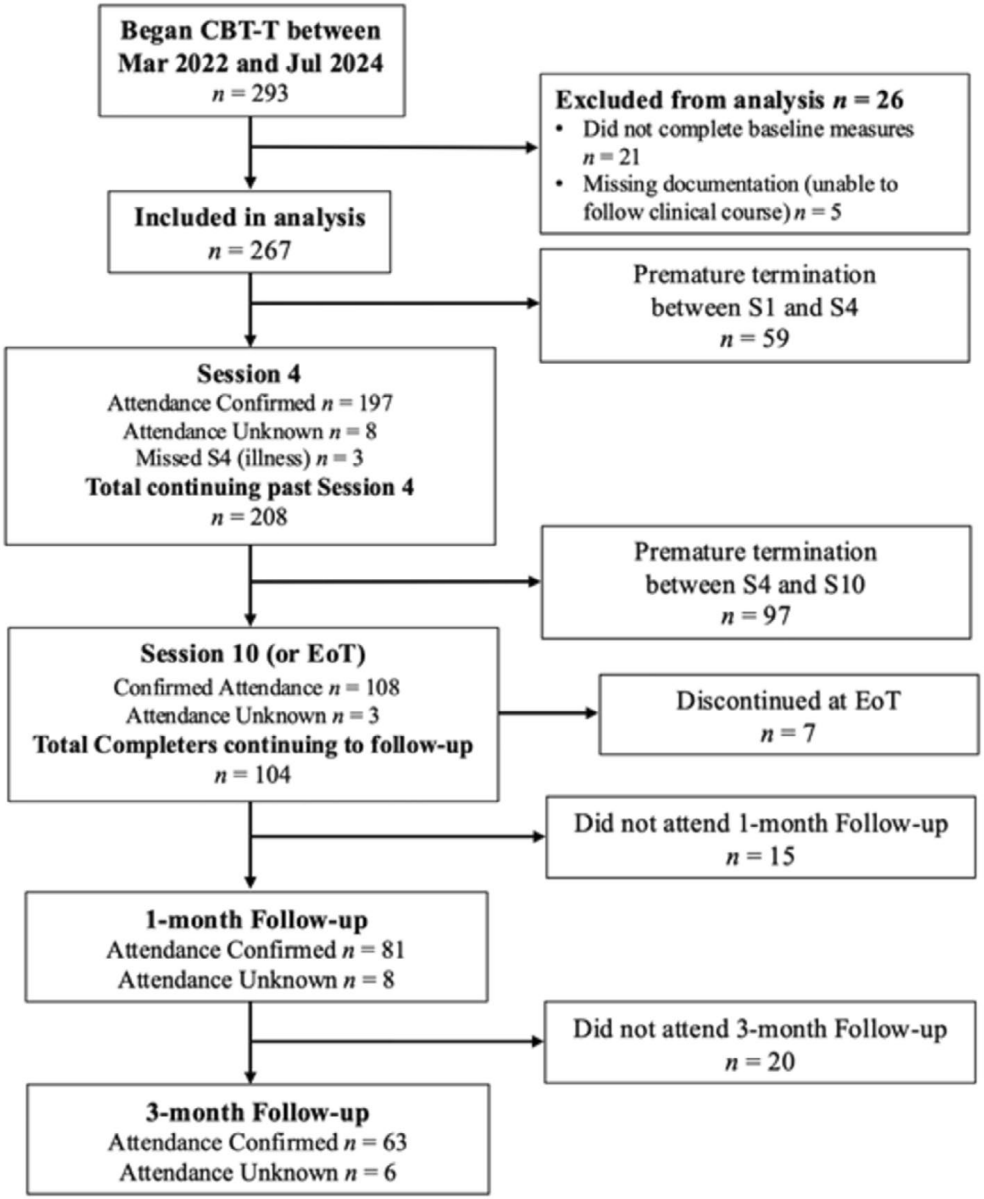
Study CONSORT diagram.

See Figure 1 for participant flow. Patients who did not complete any baseline measures prior to or within 10 days after attending Session 1 were excluded from analyses. Patients who completed at least one baseline measure were included in any analyses for which they provided data. If patients terminated treatment prematurely but continued completing questionnaires, their data were included. The final data set included 267 patients.

The NSEDPS trained 36 mental health clinicians (psychologists, social workers, nurses, occupational therapists, dietitians, and master's level therapists) through several iterations of a 10‐month CBT‐T training program. CBT‐T was delivered by NSEDPS clinicians across Nova Scotia in Community Mental Health Clinics and ED specialty clinics (located in Central and Eastern Zones), and online through the Virtual Care team. As per the CBT‐T manual (Waller et al. 2019), the standard course of treatment involved 10, 1‐hour weekly sessions followed by 1‐ and 3‐month follow‐up sessions. Since the intervention was delivered in a real‐world setting, there was inter‐clinician variability in session length and treatment duration. However, in most cases, core treatment stopped in accordance with the manual (after Session 10) or an earlier mutually agreed termination, which we defined as the end‐of‐therapy (EoT). When treatment extended beyond 10 sessions, a patient's final session before the first of two follow‐up sessions was considered EoT.

Patients completed outcome measures in the online platform, Greenspace Health, throughout treatment. Data were compiled and exported to Microsoft Excel. To establish consistency and ensure accuracy, we reviewed health records of eligible patients to determine accurate session dates/attendance.

### 2.3 Measures

#### 2.3.1 Eating Disorder Examination Questionnaire 6.0 (EDE‐Q)

The EDE‐Q is a self‐report questionnaire that measures the severity and frequency of ED cognitions and behaviors (i.e., ED psychopathology) over the past 28 days (Fairburn and Beglin 2008). It has well‐established psychometric properties (Berg et al. 2012). Items 14–15 measure the frequency of binge eating behaviors (episodes and days). Items 16–18 measure the frequency of compensatory behaviors (i.e., self‐induced vomiting, laxative use, and driven exercise). Patients completed the EDE‐Q at baseline (S1), Session 4 (S4), EoT, 1‐month follow‐up (FU1), and 3‐month follow‐up (FU3). Global EDE‐Q score was the primary outcome measure. Secondary outcomes included “abstinence” and “remission” Although there is variability in how these terms are defined across the CBT‐T literature, we selected definitions commonly used (Pellizzer et al. 2019a, 2019b, 2019c; Waller et al. 2018). Abstinence was defined as the absence of binge eating and compensatory behaviors during the 28 days prior. Remission was defined as “abstinence” plus a global score less than 1 SD above community norms (i.e., ≤ 2.77; Mond et al. 2006). Abstinence and remission were calculated at EoT, FU1, and FU3. Internal consistency was deemed good for the EDE‐Q global score (*α* = 0.88).

#### 2.3.2 Patient Health Questionnaire (PHQ‐9) and Generalized Anxiety Scale (GAD‐7)

The PHQ‐9 is a self‐report measure of depressive symptom severity (Kroenke et al. 2010), and the GAD‐7 is a self‐report measure of anxiety symptom severity (Spitzer et al. 2006). Both have well‐established psychometric properties. Patients completed these measures at S1, S4, EoT, FU1, and FU3. Internal consistency was deemed excellent for both PHQ‐9 (*α* = 0.91) and GAD‐7 (*α* = 0.92).

#### 2.3.3 Weight

Patients were weighed collaboratively with the clinician during the session. Weights for each session were extracted from patients' health records. To examine changes in weight during treatment, we clustered patients into two groups: Group A included AN, atypical anorexia nervosa (AAN), and ARFID as these disorders are characterized by significant weight loss, and weight restoration is often an important treatment target. ARFID was included in Group A as most patients (7 out of 10) in our sample with ARFID were underweight. Group B included all other EDs in the sample (BN, BED, unspecified eating disorder [UED], and purging disorder [PD]).

#### 2.3.4 Treatment Completion, Withdrawal, and Extension

“Treatment completion” was defined as completing the core 10 sessions, completing treatment in fewer than 10 sessions if treatment milestones were reached early, or completing treatment after an extension if additional sessions were offered. “Treatment withdrawal” was defined as treatment initiation followed by premature termination. As per the framework suggested by DeJong et al. (2012), withdrawal was separated into four categories: (1) clinical withdrawal: withdrawn from treatment by the clinician for reasons such as the need for more intensive treatment or for not meeting treatment nonnegotiables based on CBT‐T protocol; (2) logistical withdrawal: withdrawn due to practical or logistical reasons, such as moving away; or (3) patient‐initiated withdrawal: discontinuation of therapy without mutual agreement from the clinician, marked either by verbal discontinuation or failure to attend sessions; or (4) unknown. “Treatment extension” was defined as completing more than 10 sessions.

#### 2.3.5 Demographics and Predictor Variables

We collected demographics and clinical information (e.g., ED diagnosis) through chart review to use as potential predictors of treatment outcome, withdrawal, and extension. Potential predictors included the geographical location where CBT‐T was received (based on Nova Scotia Health's framework for categorizing the province into “zones”; i.e., Virtual, Central, Northern, Eastern, Western Zones); diagnostic categorization into Group A, and “early change” measured by the change in EDE‐Q global score from S1 to S4. Both early change in EDE‐Q and early change in ED behaviors, such as binge eating and compensatory behaviors, predict outcome in psychotherapy interventions for EDs (Chang et al. 2021). Measuring change in EDE‐Q allows for assessment of early change in transdiagnostic samples which are typical in clinical settings (Wade et al. 2025).

### 2.4 Data Analyses

Analyses were performed with R version 4.4.2. Demographics, abstinence, and remission rates were summarized descriptively. Outcome variables were assessed with mixed effects models, which included all available data from participants who completed the respective measure at baseline, under the assumption that data were missing at random. Data from participants who withdrew were included until the point of last measurement (Gabrio et al. 2022). Models were estimated within a likelihood‐based framework, which accommodates incomplete data without listwise deletion. For all analyses, an a priori two‐sided significance threshold of *α* = 0.05 was used.

#### 2.4.1 Primary and Secondary Analyses

Linear mixed effects models were used to estimate change over the course of treatment (S1, S4, EoT, FU1, and FU3) in the primary outcome, ED psychopathology measured by EDE‐Q global scores, and secondary outcomes: binge episodes and days (EDE‐Q Items 14–15), compensatory behaviors (Items 16–18), GAD‐7, PHQ‐9, and weight (for Group A and Group B). Separate models were used to assess changes in weight for Group A and Group B independently. The primary outcome model included baseline (i.e., S1) EDE‐Q scores as a covariate, time as a fixed effect, and a random intercept for participant. The model is specified as follows (in lme4 syntax): EDE‐QTotal ~ Baseline_EDE‐Q + Time + (1|Participant_ID). Secondary outcome models followed the same structure with the relevant outcome substituted in each model. All models used an unstructured covariance matrix, were estimated with restricted maximum likelihood (REML), and *p* values were obtained with Satterthwaite approximation using the *lmerTest* package (Kuznetsova et al. 2017). Model fit was assessed by comparing “null” (i.e., “Time” omitted) and “full” (i.e., “Time” included) models using likelihood ratio tests via ANOVA. Assumptions were checked graphically via the *flexplot* package (Fife, 2022). To interpret clinical significance, marginal means were estimated from the models, and effect sizes were calculated using within‐group Hedge's *g*.

#### 2.4.2 Exploratory Analyses

A linear mixed effects model following the same method as primary and secondary analyses was used to explore predictors of treatment outcome, defined as change in EDE‐Q score over the course of treatment. Predictors of treatment withdrawal and extension were assessed with generalized linear mixed effects models due to the binary nature of the data (i.e., terminated or completed, extended or not extended), with odds ratios calculated to enhance clinical interpretation. Predictor variables included: categorization into Group A, geographic zone, and baseline scores for EDE‐Q, GAD‐7, and PHQ‐9. “Early change” as a predictor was assessed only in the treatment outcome model. “Final” models for treatment outcome, withdrawal, and extension were identified via forward selection and model comparisons with ANOVA.

## 3 Results

### 3.1 Descriptive Statistics

Our sample consisted of 267 patients. Sample characteristics are described in Table 1. The average age at baseline was 30.7 years (SD = 11.50). The average number of sessions completed was 6.74 (SD = 3.59). Our sample included 24 individuals with AN, with an average BMI of 18.0 kg/m^2^ at baseline (range: 15.6–19.9 kg/m^2^). Five patients with BMIs below 17.5 kg/m^2^ were offered CBT‐T after they declined inpatient treatment. All five prematurely discontinued CBT‐T. Table 2 provides additional details regarding dropout and retention.

**TABLE 1:** Demographic characteristics of participants.

Baseline characteristic	*n*	%
Gender		
Female	234	87.6
Male	17	6.4
Non‐binary	16	6.0
Treatment delivery		
In person	136	50.9
Virtual	131	49.1
Treatment format		
Individual	244	91.4
Group	23	8.6
Location		
Central zone	121	45.7
Eastern zone	46	17.3
Virtual care zone	40	15.1
Western zone	30	11.3
Northern zone	28	10.6
Diagnosis		
Binge eating disorder^a^	72	27.0
Atypical anorexia nervosa	63	23.6
Bulimia nervosa	61	22.9
Unspecified eating disorder & purging disorder^b^	37	13.8
Anorexia nervosa	24	9.0
Avoidant/restrictive food intake disorder	10	3.7

^a^ The sample had a small number of patients (< 10) with low frequency binge eating disorder. To protect privacy, these patients were combined with full threshold binge eating disorder.

^b^ The sample had a small number of patients (< 10) with purging disorder. To protect privacy, these patients were combined with unspecified eating disorder.

**TABLE 2:** Examination of withdrawal and retention.

	*n*	%
Overall retention		
Withdrew	163	61.1
Completed	104	38.9
Withdrawal type		
Patient initiated	63	38.7
Clinician initiated	57	35.0
Unknown	34	20.9
Logistical reason	9	5.5
Withdrawal by diagnosis		
Anorexia nervosa	21	87.5
Avoidant/restrictive food intake disorder	7	70.0
Atypical anorexia nervosa	44	69.8
Bulimia nervosa	38	62.2
Unspecified eating disorder & purging disorder^a^	20	54.0
Binge eating disorder^b^	33	45.8

^a^ The sample had a small number of patients (< 10) with purging disorder. To protect privacy, these patients were combined with unspecified eating disorder.

^b^ The sample had a small number of patients (< 10) with low frequency BED. To protect privacy, these patients were combined with full threshold BED.

### 3.2 Primary and Secondary Analyses

**FIGURE 2:**
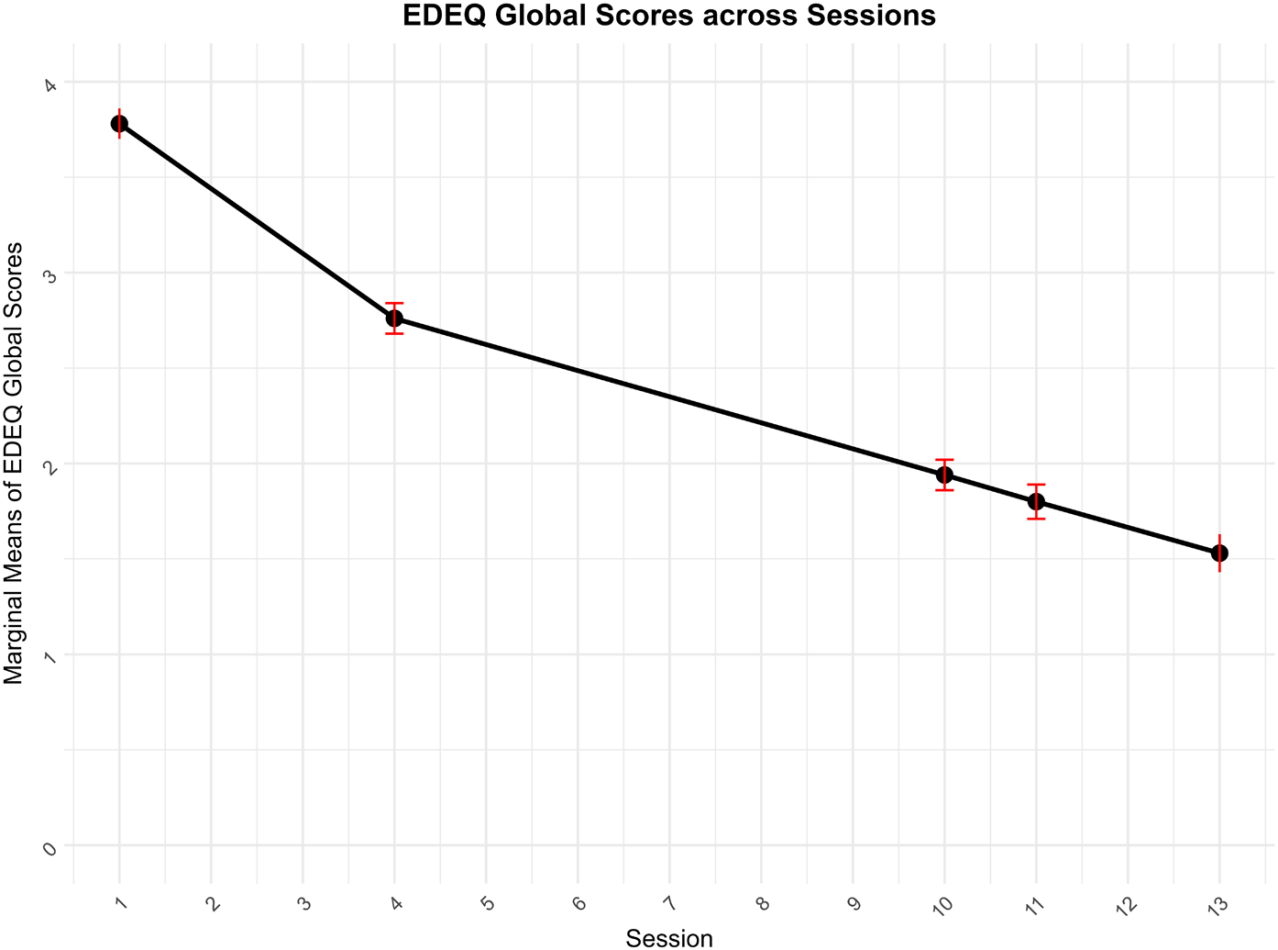
Change in EDE‐Q global scores across time. EDE‐Q: Eating disorder examination questionnaire error bars represent 95% confidence intervals of the estimated marginal means.

There was a significant effect of time on ED psychopathology, with EDE‐Q global scores decreasing across time (see Figure 2). Effect sizes were very large at EoT (*g* = −1.60) and FU3 (*g* = −1.90). Model properties and marginal means from all study measures are described in Tables 3 and 4, respectively.

**TABLE 3:** Model properties of primary and secondary outcome measures across time.

	*β*	95% CI	*p* ^c^
EDE‐Q global	−0.14	[−0.15, −0.13]	**< 0.001**
Eating disorder symptoms			
Binge episodes	−0.28	[−0.32, −0.24]	**< 0.001**
Binge days	−0.27	[−0.31, −0.23]	**< 0.001**
Vomiting episodes	−0.36	[−0.48, −0.24]	**0.004**
Laxative episodes	−0.001	[−0.04, 0.04]	0.999
Driven exercise episodes	−0.36	[−0.45, −0.27]	**< 0.001**
PHQ‐9	−0.31	[−0.36, −0.26]	**< 0.001**
GAD‐7	−0.26	[−0.31, −0.21]	**< 0.001**
Weight by diagnostic categorization			
Group A^a^	0.28	[0.26, 0.30]	**< 0.001**.
Group B^b^	−0.01	[−0.05, 0.03]	0.775

Abbreviations: CI, confidence interval; EDE‐Q, eating disorder examination questionnaire; GAD‐7, general anxiety disorder questionnaire; PHQ‐9, patient health questionnaire.

^a^ Group A included anorexia nervosa, atypical anorexia, and avoidant restrictive food intake disorder.

^b^ Group B included bulimia nervosa, binge eating disorders, purging disorder, and unspecified eating disorders.

^c^ Bold values indicates the significance level defined as *p* ≤ 0.05.

**TABLE 4:** Marginal means and standard deviations of linear mixed effects models evaluating study measures.

Measure	S1	S4	S10	FU1	FU3
	*M* 95% CI	*M* 95% CI	*M* 95% CI	*M* 95% CI	*M* 95% CI
EDE‐Q Global	3.78 [3.70, 3.86]	2.76 [2.60, 2.92]	1.94 [1.78, 2.10]	1.80 [1.63, 1.98]	1.53 [1.33, 1.73]
Eating disorder symptoms					
Binge episodes	12.28 [11.57, 12.99]	3.72 [3.06, 4.38]	2.05 [1.40, 2.69]	1.77 [1.10, 2.44]	1.21 [0.46, 1.96]
Binge days	11.37 [10.80, 11.94]	3.48 [2.88, 4.07]	1.85 [1.28, 2.43]	1.58 [0.97, 2.20]	1.04 [0.35, 1.74]
Vomiting episodes	10.73 [9.43, 12.03]	3.50 [2.26, 4.74]	1.34 [0.23, 2.44]	0.98 [−0.28, 2.23]	0.26 [−1.38, 1.89]
Driven exercise episodes	11.56 [10.57, 12.55]	3.85 [2.85, 4.86]	1.71 [0.73, 2.68]	1.35 [0.27, 2.42]	0.63 [−0.68, 1.95]
GAD‐7	13.21 [12.88, 13.54]	10.58 [9.92, 11.24]	9.01 [8.34, 9.68]	8.75 [8.03, 9.47]	8.22 [7.38, 9.07]
PHQ‐9	15.10 [14.73, 15.47]	11.66 [10.97, 12.35]	9.80 [9.10, 10.50]	9.49 [8.74, 10.24]	8.87 [7.97, 9.76]

Abbreviations: EDE‐Q, eating disorder examination questionnaire; GAD‐7, general anxiety disorder questionnaire; PHQ‐9, patient health questionnaire.

**FIGURE 3:**
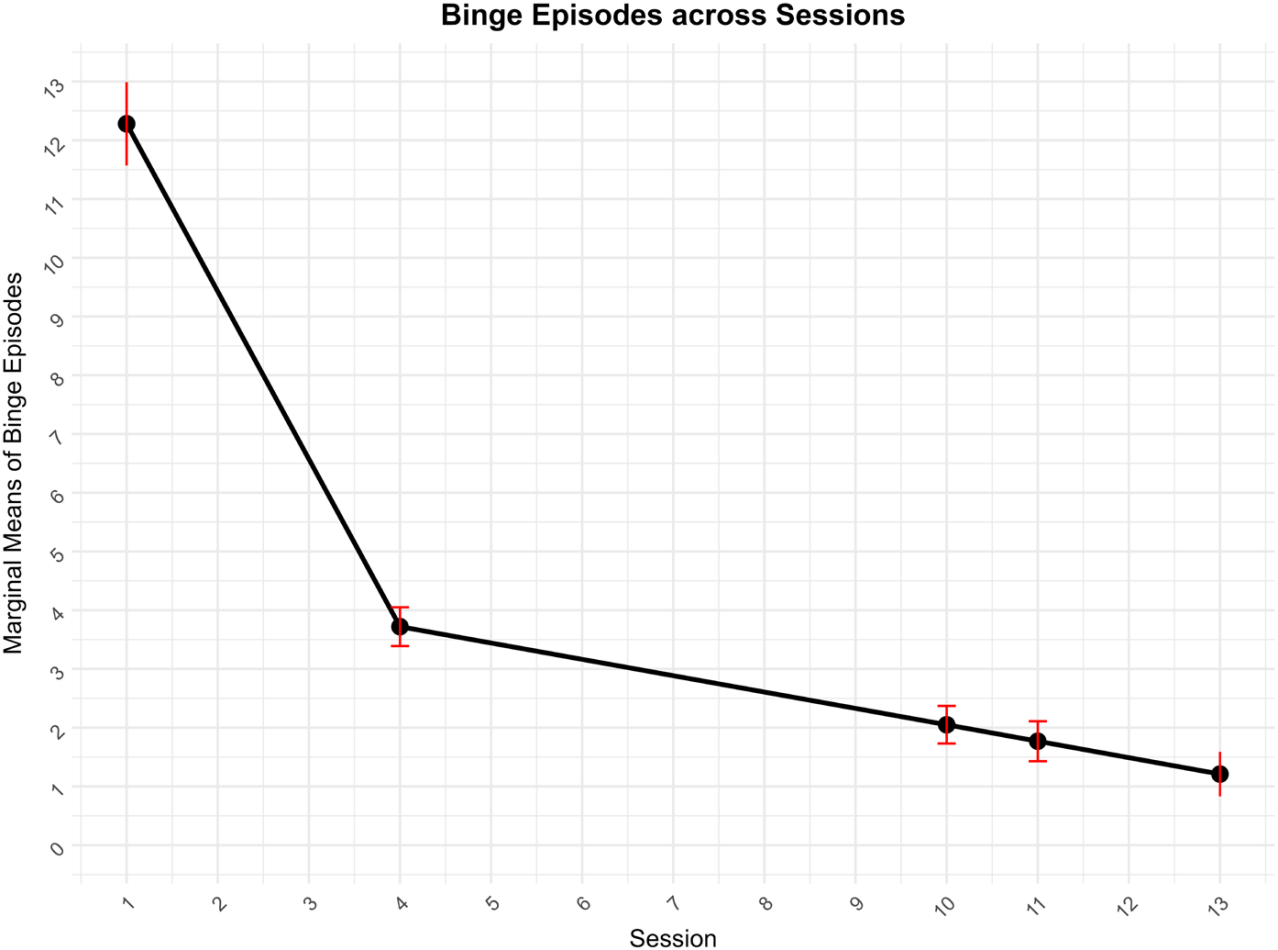
Change in binge‐episode frequency across time. Error bars represent 95% confidence intervals of the estimated marginal means.

There was a significant effect of time on the frequency of binge episodes (see Figure 3), binge days, vomiting episodes, and driven exercise episodes, with these behaviors all decreasing across time. There was no significant effect of time on the frequency of laxative use, likely due to low frequency at baseline within our sample. Effect sizes were very large for binge‐episode frequency at EoT (*g* = −1.20) and FU3 (*g* = −1.27) and large for vomiting at EoT (*g* = −0.99) and FU3 (*g* = −1.05).

There was a significant effect of time on depression and anxiety symptom severity, with PHQ‐9 and GAD‐7 scores decreasing across time. Effect sizes were large at EoT (*g* = −1.02 and *g* = −0.88, respectively).

There was a significant effect of time on weight for those categorized into Group A, whereby weight increased over time. There was no significant effect of time on weight for Group B.

### 3.3 Abstinence and Remission Rates

At EoT, 98 participants completed an EDE‐Q; 50% (*n* = 45) achieved abstinence and 37% (*n =* 36) achieved remission. At FU1, 78 participants completed an EDE‐Q; 53% (*n* = 41) achieved abstinence and 46% (*n* = 36) achieved remission. At FU3, 55 participants completed an EDE‐Q; 56% (*n* = 31) achieved abstinence and 53% (*n* = 29) achieved remission.

### 3.4 Exploratory Analyses

#### 3.4.1 Treatment Outcome

The final model for treatment outcome contained baseline GAD‐7, baseline PHQ‐9, and early change (see Table 5 for model properties of predictor analyses). Early change between S1 and S4 was associated with lower EDE‐Q global scores throughout treatment. Baseline GAD‐7 and PHQ‐9 were not significant predictors.

**TABLE 5:** Model properties for secondary predictor analyses.

	*β*	95% CI	*p* c	OR^a^
Treatment outcome				
Baseline GAD‐7	−0.01	[0.00, 0.02]	0.868	N/A
Baseline PHQ‐9	−0.002	[−0.01, 0.01]	0.217	N/A
Early change	−0.88	[−0.92, −0.82]	**< 0.001**	N/A
Treatment withdrawal				
Group A^b^	0.99	[0.69, 1.29]	**0.001**	2.69
Baseline PHQ‐9	0.04	[0.02, 0.06]	0.098	N/A
Treatment extension				
Group A^b^	−1.12	[−1.68, −0.56]	**0.044**	0.33
Central zone	0.40	[−0.44, 1.24]	0.634	1.49
Northern zone	2.43	[1.51, 3.35]	**0.008**	11.36
Eastern zone	0.75	[−0.32, 1.82]	0.484	2.12
Western zone	1.98	[1.13, 2.83]	**0.020**	7.24
Baseline EDE‐Q	0.73	[0.46, 0.99]	**0.006**	N/A
Baseline PHQ‐9	−0.07	[−0.11, −0.03]	0.119	N/A

Abbreviations: CI, confidence interval; ED, eating disorder; EDE‐Q, eating disorder examination questionnaire; GAD‐7, general anxiety disorder questionnaire; OR, odds ratio; PHQ‐9, patient health questionnaire.

^a^ Odds ratios are only presented for categorical variables.

^b^ Group A included patients with anorexia nervosa, atypical anorexia, and avoidant restrictive food intake disorder.

^c^ Bold values indicates the significance level defined as *p* ≤ 0.05.

#### 3.4.2 Treatment Withdrawal

The final model for treatment withdrawal contained baseline PHQ‐9 and categorization into Group A. Baseline PHQ‐9 was not a significant predictor of withdrawal. Categorization into Group A was significant, indicating that those with a diagnosis of AN, AAN, or ARFID were more likely to prematurely terminate treatment.

#### 3.4.3 Treatment Extension

Despite a core feature of CBT‐T being the 10‐session duration, some patients received longer treatment. In those with available data (*n* = 266), 28 patients received additional sessions, ranging from 11 to 16 total sessions. Most patients received 1–2 additional sessions (*n* = 20). Of the patients receiving extended treatment, over 50% (*n* = 18) were treated by the same four clinicians.

The final model for treatment extension contained the variables of geographic zone, categorization into Group A, and baseline PHQ‐9. The variables geographic zone, categorization into Group A, and baseline EDE‐Q were significant predictors of treatment extension. Individuals with AN, AAN, or ARFID were significantly less likely to receive extended treatment compared to individuals with other diagnoses. In contrast, patients in the Northern Zone and Eastern Zone were significantly more likely to receive extended treatment compared to the Virtual Zone. Baseline EDE‐Q also significantly predicted treatment extension, whereby those with higher scores were more likely to receive extended treatment. Baseline PHQ‐9 was not a significant predictor of extension.

To examine whether receiving longer treatment affected treatment outcome, we conducted an additional analysis whereby we repeated the primary analysis while including treatment extension within the model. Despite receiving additional sessions, there was no significant difference in treatment outcome for those who had extended treatment compared to those who did not (*β* = 0.37, *p* = 0.095, CI [0.15, 0.59]).

### 3.5 Sensitivity Analysis

A sensitivity analysis using multiple imputation was performed using the “mice” package (van Buuren and Groothuis‐Oudshoorn 2011) for primary and secondary analyses. Results were consistent with nonimputed models, with no meaningful changes in direction, magnitude, or significance. Full results are included as Supporting Information.

## 4 Discussion

This retrospective study examined the outcomes of CBT‐T for ED patients seeking publicly funded treatment in Nova Scotia, Canada following the implementation of a large‐scale CBT‐T training program.

### 4.1 Eating Disorder Psychopathology

Patients experienced reduced ED psychopathology as measured by the global score of the EDE‐Q. Improvements were maintained at follow‐up. Reductions corresponded to very large effect sizes at EoT and FU3. This is consistent with results from meta‐analyses by Keegan et al. (2022) and Öst et al. (2024), which found CBT‐T and CBT‐ED, respectively, were associated with large reductions in ED psychopathology at EoT and follow‐up.

### 4.2 Eating Disorder Symptomatology

Patients experienced reductions in binge eating and compensatory behaviors during treatment whereby binge eating episodes in the past 28 days decreased from an average of 12 at baseline to an average of 1–2 episodes in the 28 days prior to EoT, FU1, and FU3. These changes corresponded to very large effect size reductions. Reductions in eating disorder symptoms including self‐induced vomiting and driven exercise were also observed at EoT, FU1, and FU3. Reductions in binge eating and self‐induced vomiting frequency are consistent with those reported by Keegan et al. (2022).

### 4.3 Remission and Abstinence

When defining abstinence as total cessation of binge and/or compensatory behaviors during the 28 days prior to EoT, 50% of completers with available data achieved abstinence. At 1‐ and 3‐month follow‐up, abstinence rates for completers were 53% and 46%, respectively. Several studies examining outcomes of CBT‐T using the same definition of abstinence reported abstinence rates for completers at EoT ranging from 30% to 77% (Pellizzer et al. 2019a, 2019b, 2019c; Rose et al. 2021). At 1‐ and 3‐month follow‐up, abstinence rates for completers ranged from 56% to 62% (Pellizzer et al. 2019a, 2019b, 2019c). Thus, our abstinence rates, particularly at 3‐month follow‐up, are somewhat lower than those reported elsewhere.

At EoT, 37% of completers met criteria for remission (defined as “Abstinence” plus EDE‐Q global ≤ 2.77). At 1‐ and 3‐month follow‐up, remission rates were 56% and 53%, respectively. Other studies of CBT‐T using the same definition of remission reported remission rates for completers at EoT ranged from 23% to 54% (Pellizzer et al. 2019a, 2019b, 2019c; Rose et al. 2021). At 1‐ and 3‐month follow‐up, remission rates for completers ranged from 38% to 50% and 46% to 63% (Pellizzer et al. 2019a, 2019b, 2019c). Remission and abstinence rates should be interpreted in the context of our study's dropout rate (61%), which is higher than the 35%–50% dropout rates reported in studies discussed above.

### 4.4 Depression and Anxiety

Treatment was associated with reduced anxiety and depression. Reductions corresponded to large/very large effect sizes at EoT. These effect sizes are higher than those reported in the meta‐analysis by Keegan et al. (2022) where medium effect size reductions were observed for anxiety and large reductions for depression.

### 4.5 Predictors of Outcome, Withdrawal, and Extension

#### 4.5.1 Treatment Outcome

Early change was the only significant predictor of treatment outcome, indicating that greater reduction in EDE‐Q in the first four sessions was associated with lower EDE‐Q at EoT and FU. This finding is consistent with previous research, which has shown that early change in ED psychopathology is a predictor of outcome during psychotherapy (Chang et al. 2021), and CBT‐T specifically (Keegan et al. 2022). Early change in behavioral features such as binge eating, weight, and compensatory behaviors also predicts positive outcomes in psychotherapy for EDs (Chang et al. 2021). More recently, Gatley et al. (2024) sought to examine whether baseline characteristics such as diagnosis type, wait time, depression, anxiety, and therapeutic alliance predicted early change in EDE‐Q scores during CBT‐T. However, no significant interactions were found (Gatley et al. 2024).

#### 4.5.2 Treatment Withdrawal

We observed a higher rate of dropout than existing studies of CBT‐T. For example, a review by Paphiti and Newman (2023) found the attrition rate of studies on CBT‐T ranged from 23% to 50%. A potential explanation for our higher dropout rates is the difference in our sample composition compared to previous research. When examining the studies included in the review by Paphiti and Newman (2023) the percentage of the samples meeting criteria for AAN or AN ranged from 0% to 12% in studies that specified diagnosis (Gatley et al. 2024; Pellizzer et al. 2019a, 2019b; Rose et al. 2021; Tatham et al. 2020; Wade et al. 2021; Waller et al. 2018). In contrast, over 30% of our sample met criteria for AN or AAN. This differential is relevant considering we observed that 75% of patients with AN or AAN dropped out compared to 53% of individuals with BN and BED, which is only slightly higher than existing data on CBT‐T.

Since patients with a diagnosis of AN, AAN, and ARFID were almost three times more likely to drop out compared to other diagnoses, it is important to consider the possible reasons for this inter‐diagnosis variability. AN (with BMI > 17.5) and AAN, both of which are included as nonunderweight disorders in the CBT‐T manual, share the characteristic of weight suppression, which is the difference between an individual's highest adult weight (excluding pregnancy) and current weight (Lowe et al. 2018). Since weight‐suppressed patients tend to gain more weight overall, and at a faster rate, during ED treatment (Carter et al. 2015), it is possible that in CBT‐T they may disengage from treatment or struggle to achieve expected progress by Session 4 due to anxiety regarding weight regain. In support of this hypothesis, in our sample, 75% of patients with AN or AAN discontinued treatment prematurely, completing on average fewer than five sessions. In future research, it will be important to consider how to increase engagement early in treatment and improve retention for patients with AN and AAN while still offering shorter treatment durations than CBT‐ED. Future research should also explore weight suppression as a predictor of outcome and retention in CBT‐ED.

Another potential explanation for our higher dropout rate is differences in therapist characteristics compared to previous studies. Although most of the published studies on CBT‐T have also been conducted within real‐world clinical settings, most had fewer than seven therapists. These therapists were often described as trainees who received regular (weekly/biweekly) supervision by clinicians who authored the CBT‐T treatment manual. In contrast, we had 36 different therapists with diverse backgrounds offering CBT‐T across our province. Although we utilized a training program (16 hours of didactic training) and offered 8 months of mandatory weekly supervision followed by optional ongoing peer supervision, there was less stringent *long‐term* supervision. This may have impacted adherence to the treatment protocol and ultimately impacted treatment retention rates. Presently, we do not utilize a measure of therapist fidelity or track participation in ongoing supervision within our service (beyond the initial 8‐month mandatory supervision period), nor do we utilize “supervision of supervisors”, all of which could be beneficial additions to examine these factors going forward.

#### 4.5.3 Treatment Extension

Overall, treatment extension beyond the core 10 sessions of CBT‐T was relatively infrequent. Those with AN, AAN, and ARFID were roughly one‐third as likely to extend treatment compared to those with other diagnoses. This could be explained by increased dropout among the former, which eliminated the chance to extend treatment altogether. We also found that patients with higher EDE‐Q scores at Session 1, and those located in specific geographic zones, were more likely to receive extended treatment. The zonal variations were accounted for by a small number of individual clinicians within those zones who were more likely to extend treatment. Based on our finding that higher EDE‐Q at Session 1 predicted treatment extension, it is possible these few clinicians extended treatment because they assumed longer treatment would benefit patients who have more severe symptoms. Although this assumption has merit, patients who extended treatment did not have better outcomes at EoT when compared to those that only received 10 sessions.

#### 4.5.4 Strengths and Limitations

This study has several strengths, including the use of a large, transdiagnostic, and naturalistic sample of patients and clinicians. Moreover, we offer the first demonstration of outcomes from CBT‐T when implemented in a publicly funded North American healthcare setting following a provincial training program. Our results contribute to the literature, demonstrating that CBT‐T is associated with reduced ED psychopathology and symptomatology that are comparable to other forms of CBT‐ED despite its shorter duration. We have also examined a variety of predictors to explore factors that may influence treatment outcomes, withdrawal, and extension.

We acknowledge several limitations with the current study. First, roughly 47% of the data were missing. Our high rate of missing data is partially explained by our dropout rate, as the percentage of missing data includes data missing due to dropout. A recent publication examining outcomes from 54 sites in England offering ED treatment including CBT‐T reported the percentage of missing data at baseline ranged from 35% to 61%, and 80% to 90% at post‐treatment (Allen et al. 2024). We accounted for missing data with linear mixed effects models and a sensitivity analysis using multiple imputation. Despite the use of data analysis methods to reduce bias, it is possible patients who withdrew may have experienced worse outcomes than those who completed. Second, the lack of formal assessment of clinician treatment fidelity does not allow us to comment on adherence to the CBT‐T protocol. Organizations interested in implementing CBT‐T within their service should consider building ongoing clinical supervision and routine assessment of treatment fidelity into their programs. Third, our sample of patients was largely composed of women, with a small representation of men and nonbinary individuals. We also did not collect race‐related information. Thus, it is not clear whether our results would generalize to all genders and races.

## 5 Conclusions

CBT‐T is a promising, scalable treatment for both specialized ED and nonspecialized services. Dissemination of CBT‐T provincially allowed patients to access eating disorder care in their home communities and was associated temporally with reductions in wait times for assessment and treatment. Future research could explore evidence‐based adaptations to CBT‐T that enhance retention of individuals with AN/AAN. Additionally, it would be valuable to investigate the role of weight suppression, evaluate the use of CBT‐T in more diverse populations, and assess clinician treatment fidelity within real‐world clinical settings.

## Author Contributions

**Laura Dixon:** writing – original draft, investigation, methodology, writing – review and editing, formal analysis, data curation, project administration, validation, software, visualization. **Colby Price:** investigation, formal analysis, writing – original draft, writing – review and editing, methodology, data curation, project administration, validation, software, visualization. **Sara Bartel:** writing – review and editing, writing – original draft, visualization. **Anastasia Harris:** writing – review and editing, formal analysis, data curation, methodology, investigation, software, validation. **Marika Schenkels:** investigation, writing – original draft, methodology, data curation, software. **Toni Spinella:** writing – review and editing, formal analysis, software, validation. **Abraham Nunes:** formal analysis, writing – review and editing, software, validation. **Sarrah I. Ali:** writing – review and editing, formal analysis, validation. **Glenn Waller:** writing – review and editing. **Jessica Wournell:** writing – review and editing. **Susan Gamberg:** writing – review and editing. **Aaron Keshen:** supervision, writing – review and editing, writing – original draft, conceptualization, methodology, project administration, investigation, resources, visualization, validation.

## Conflicts of Interest

G.W. is an author of the CBT‐T manual cited in this work and receives royalties from its publishers. The other authors declare no conflicts of interest.

## Supporting information


**Data S1.** eat24498‐sup‐0001‐supinfo.

## Data Availability

The data that support the findings of this study are available on request from the corresponding author. The data are not publicly available due to privacy or ethical restrictions.
