# Supplementary material for: Delivering Brief Cognitive Behavioral Therapy (CBT‐T) for Eating Disorders: Examining Real‐World Outcomes of a Large‐Scale Training Program
Source: Int J Eat Disord. 2025 Jul 2;58(10):1946–56. doi: 10.1002/eat.24498 (PMC12501551; doi:10.1002/eat.24498)
Supplement: Supplementary file 1 — Data S1. eat24498‐sup‐0001‐supinfo. [file EAT-58-1946-s001.docx]

| **EDEQ Model Comparisons** | | | | | | |
| --- | --- | --- | --- | --- | --- | --- |
|  | **Null Model** | | | **Full Model** | | |
| *Predictors* | *Estimates* | *95% CI* | *p* | *Estimates* | *95% CI* | *p* |
| (Intercept) | -0.16 | [-0.43, 0.11] | 0.556 | 0.71 | [0.45, 0.97] | **0.006** |
| Base EDE-Q | 0.65 | [0.58, 0.72] | **<0.001** | 0.68 | [0.67, 0.69] | **<0.001** |
| Session |  |  |  | -0.14 | [-0.15, -0.13] | **<0.001** |
| **Random Effects** | | | | | | |
| σ^2^ | 0.68 | | | 0.41 | | |
| τ_00_ | 0.70 _Patient_ID_ | | | 0.72 _Patient_ID_ | | |
| ICC | 0.51 | | | 0.64 | | |
| N | 169 _Patient_ID_ | | | 169 _Patient_ID_ | | |
| Observations | 369 | | | 369 | | |
| Marginal R^2^ / Conditional R^2^ | 0.286 / 0.647 | | | 0.419 / 0.789 | | |
| AIC | 1026.31 | | | **903.48** | | |
| BIC | 1041.49 | | | 922.69 | | |

*Note.* EDE-Q = Eating Disorder Examination Questionnaire; CI = Confidence Interval; ICC = Intraclass Correlation Coefficient; AIC = Akaike Information Criterion; BIC = Bayesian Information Criterion.

| **Binge Episodes Model Comparisons** | | | | | | |
| --- | --- | --- | --- | --- | --- | --- |
|  | **Null Model** | | | **Full Model** | | |
| *Predictors* | *Estimates* | *95% CI* | *p* | *Estimates* | *95% CI* | *p* |
| (Intercept) | 2.26 | [1.73, 2.79] | **<0.001** | 4.23 | [3.66, 4.80] | **<0.001** |
| Base 14 | 0.04 | [0.01, 0.07] | 0.219 | 0.05 | [0.02, 0.09] | 0.138 |
| Session |  |  |  | -0.28 | [-0.32, -0.24] | **<0.001** |
| **Random Effects** | | | | | | |
| σ^2^ | 5.33 | | | 4.42 | | |
| τ_00_ | 10.10 _Patient_ID_ | | | 9.31 _Patient_ID_ | | |
| ICC | 0.65 | | | 0.68 | | |
| N | 130 _Patient_ID_ | | | 130 _Patient_ID_ | | |
| Observations | 299 | | | 299 | | |
| Marginal R^2^ / Conditional R^2^ | 0.011 / 0.658 | | | 0.079 / 0.704 | | |
| AIC | 1448.20 | | | **1405.70** | | |
| BIC | 1462.70 | | | 1423.80 | | |

*Note.* CI = Confidence Interval; ICC = Intraclass Correlation Coefficient; AIC = Akaike Information Criterion; BIC = Bayesian Information Criterion.

| **Binge Days Model Comparisons** | | | | | | |
| --- | --- | --- | --- | --- | --- | --- |
|  | **Null Model** | | | **Full Model** | | |
| *Predictors* | *Estimates* | *95% CI* | *p* | *Estimates* | *95% CI* | *p* |
| (Intercept) | 1.80 | [1.30, 2.30] | **<0.001** | 3.72 | [3.17, 4.27] | **<0.001** |
| Base 15 | 0.06 | [0.03, 0.09] | 0.075 | 0.07 | [-0.48, 0.62] | **0.038** |
| Session |  |  |  | -0.27 | [-0.31, -0.23] | **<0.001** |
| **Random Effects** | | | | | | |
| σ^2^ | 5.62 | | | 4.67 | | |
| τ_00_ | 7.23 _Patient_ID_ | | | 6.98 _Patient_ID_ | | |
| ICC | 0.56 | | | 0.60 | | |
| N | 132 _Patient_ID_ | | | 132 _Patient_ID_ | | |
| Observations | 305 | | | 305 | | |
| Marginal R^2^ / Conditional R^2^ | 0.020 / 0.572 | | | 0.095 / 0.637 | | |
| AIC | 1461.30 | | | **1424.00** | | |
| BIC | 1475.80 | | | 1442.20 | | |

*Note.* CI = Confidence Interval; ICC = Intraclass Correlation Coefficient; AIC = Akaike Information Criterion; BIC = Bayesian Information Criterion.

| **Vomiting Model Comparisons** | | | | | | |
| --- | --- | --- | --- | --- | --- | --- |
|  | **Null Model** | | | **Full Model** | | |
| *Predictors* | *Estimates* | *95% CI* | *p* | *Estimates* | *95% CI* | *p* |
| (Intercept) | 0.26 | [-0.38, 0.90] | 0.684 | 3.16 | [2.02, 4.30] | **0.007** |
| Base 16 | 0.19 | [0.14, 0.24] | **<0.001** | 0.18 | [0.14, 0.22] | **<0.001** |
| Session |  |  |  | -0.36 | [-0.48, -0.24] | **0.004** |
| **Random Effects** | | | | | | |
| σ^2^ | 16.85 | | | 15.20 | | |
| τ_00_ | 0.00 _Patient_ID_ | | | 0.00 _Patient_ID_ | | |
| N | 43 _Patient_ID_ | | | 43 _Patient_ID_ | | |
| Observations | 83 | | | 83 | | |
| Marginal R^2^ / Conditional R^2^ | 0.184 / NA | | | 0.265 / NA | | |
| AIC | 419.62 | | | **414.04** | | |
| BIC | 428.67 | | | 425.35 | | |

*Note.* CI = Confidence Interval; ICC = Intraclass Correlation Coefficient; AIC = Akaike Information Criterion; BIC = Bayesian Information Criterion.

| **Laxative Abuse Model Comparisons** | | | | | | |
| --- | --- | --- | --- | --- | --- | --- |
|  | **Null Model** | | | **Full Model** | | |
| *Predictors* | *Estimates* | *95% CI* | *p* | *Estimates* | *95% CI* | *p* |
| (Intercept) | -0.53 | [-1.32, 0.26] | 0.508 | -0.52 | [-1.36, 0.32] | 0.540 |
| Base 17 | 0.26 | [0.18, 0.34] | **0.003** | 0.26 | [0.18, 0.34] | **0.003** |
| Session |  |  |  | -0.00 | [-0.04, 0.04] | 0.990 |
| **Random Effects** | | | | | | |
| σ^2^ | 0.36 | | | 0.36 | | |
| τ_00_ | 3.76 _Patient_ID_ | | | 3.76 _Patient_ID_ | | |
| ICC | 0.91 | | | 0.91 | | |
| N | 14 _Patient_ID_ | | | 14 _Patient_ID_ | | |
| Observations | 27 | | | 27 | | |
| Marginal R^2^ / Conditional R^2^ | 0.375 / 0.945 | | | 0.375 / 0.945 | | |
| AIC | **97.73** | | | 99.73 | | |
| BIC | 102.92 | | | 106.21 | | |

*Note.* CI = Confidence Interval; ICC = Intraclass Correlation Coefficient; AIC = Akaike Information Criterion; BIC = Bayesian Information Criterion.

| **Driven Exercise Model Comparisons** | | | | | | |
| --- | --- | --- | --- | --- | --- | --- |
|  | **Null Model** | | | **Full Model** | | |
| *Predictors* | *Estimates* | *95% CI* | *p* | *Estimates* | *95% CI* | *p* |
| (Intercept) | -0.71 | [-1.39, -0.03] | 0.295 | 2.02 | [1.08, 2.96] | **0.033** |
| Base 18 | 0.28 | [0.24, 0.32] | **<0.001** | 0.27 | [0.23, 0.31] | **<0.001** |
| Session |  |  |  | -0.36 | [-0.45, -0.27] | **<0.001** |
| **Random Effects** | | | | | | |
| σ^2^ | 14.63 | | | 12.08 | | |
| τ_00_ | 3.98 _Patient_ID_ | | | 5.34 _Patient_ID_ | | |
| ICC | 0.21 | | | 0.31 | | |
| N | 70 _Patient_ID_ | | | 70 _Patient_ID_ | | |
| Observations | 150 | | | 150 | | |
| Marginal R^2^ / Conditional R^2^ | 0.249 / 0.409 | | | 0.307 / 0.519 | | |
| AIC | 826.37 | | | **814.96** | | |
| BIC | 838.19 | | | 829.74 | | |

*Note.* CI = Confidence Interval; ICC = Intraclass Correlation Coefficient; AIC = Akaike Information Criterion; BIC = Bayesian Information Criterion.

| **GAD-7 Model Comparisons** | | | | | | |
| --- | --- | --- | --- | --- | --- | --- |
|  | **Null Model** | | | **Full Model** | | |
| *Predictors* | *Estimates* | *95% CI* | *p* | *Estimates* | *95% CI* | *p* |
| (Intercept) | 0.63 | [-0.16, 1.42] | 0.427 | 2.43 | [1.58, 3.28] | **0.004** |
| Base GAD-7 | 0.72 | [0.66, 0.78] | **<0.001** | 0.72 | [0.66, 0.78] | **<0.001** |
| Session |  |  |  | -0.26 | [-0.31, -0.26] | **<0.001** |
| **Random Effects** | | | | | | |
| σ^2^ | 9.39 | | | 8.52 | | |
| τ_00_ | 10.13 _Patient_ID_ | | | 9.89 _Patient_ID_ | | |
| ICC | 0.52 | | | 0.54 | | |
| N | 168 _Patient_ID_ | | | 168 _Patient_ID_ | | |
| Observations | 355 | | | 355 | | |
| Marginal R^2^ / Conditional R^2^ | 0.426 / 0.724 | | | 0.455 / 0.748 | | |
| AIC | 1996.90 | | | **1972.60** | | |
| BIC | 2012.40 | | | 1991.90 | | |

*Note.* GAD-7 = Generalized Anxiety Scale; CI = Confidence Interval; ICC = Intraclass Correlation Coefficient; AIC = Akaike Information Criterion; BIC = Bayesian Information Criterion.

| **PHQ-9 Model Comparisons** | | | | | | |
| --- | --- | --- | --- | --- | --- | --- |
|  | **Null Model** | | | **Full Model** | | |
| *Predictors* | *Estimates* | *95% CI* | *p* | *Estimates* | *95% CI* | *p* |
| (Intercept) | 0.38 | [-0.47, 1.23] | 0.653 | 2.55 | [1.64, 3.46] | **0.005** |
| Base PHQ-9 | 0.70 | [0.65, 0.75] | **<0.001** | 0.70 | [0.65, 0.75] | **<0.001** |
| Session |  |  |  | -0.31 | [-0.36, -0.27] | **<0.001** |
| **Random Effects** | | | | | | |
| σ^2^ | 11.70 | | | 10.45 | | |
| τ_00_ | 10.30 _Patient_ID_ | | | 10.08 _Patient_ID_ | | |
| ICC | 0.47 | | | 0.49 | | |
| N | 168 _Patient_ID_ | | | 168 _Patient_ID_ | | |
| Observations | 356 | | | 356 | | |
| Marginal R^2^ / Conditional R^2^ | 0.435 / 0.700 | | | 0.471 / 0.731 | | |
| AIC | 2059.80 | | | **2030.80** | | |
| BIC | 2075.30 | | | 2050.20 | | |

*Note.* PHQ-9 = Patient Health Questionnaire CI = Confidence Interval; ICC = Intraclass Correlation Coefficient; AIC = Akaike Information Criterion; BIC = Bayesian Information Criterion.

| **Weight Model Comparisons** | | | | | | |
| --- | --- | --- | --- | --- | --- | --- |
|  | **Null Model** | | | **Full Model** | | |
| *Predictors* | *Estimates* | *95% CI* | *p* | *Estimates* | *95% CI* | *p* |
| (Intercept) | 2.30 | [1.79, 2.81] | **<0.001** | 1.97 | [1.41, 2.53] | **<0.001** |
| Base Weight | 0.98 | [0.38, 1.58] | **<0.001** | 0.98 | [0.95, 1.01] | **<0.001** |
| Session |  |  |  | 0.08 | [0.05, 0.11] | **0.010** |
| **Random Effects** | | | | | | |
| σ^2^ | 8.64 | | | 8.60 | | |
| τ_00_ | 7.24 _Patient_ID_ | | | 7.20 _Patient_ID_ | | |
| ICC | 0.46 | | | 0.46 | | |
| N | 227 _Patient_ID_ | | | 227 _Patient_ID_ | | |
| Observations | 1335 | | | 1335 | | |
| Marginal R^2^ / Conditional R^2^ | 0.986 / 0.992 | | | 0.986 / 0.992 | | |
| AIC | 7046.472 | | | **7041.776** | | |
| BIC | 7067.259 | | | 7067.760 | | |

*Note.* CI = Confidence Interval; ICC = Intraclass Correlation Coefficient; AIC = Akaike Information Criterion; BIC = Bayesian Information Criterion.

| **Weight (of Restrictive EDs) Model Comparisons** | | | | | | |
| --- | --- | --- | --- | --- | --- | --- |
|  | **Null Model** | | | **Full Model** | | |
| *Predictors* | *Estimates* | *95% CI* | *p* | *Estimates* | *95% CI* | *p* |
| (Intercept) | 3.41 | [1.81, 5.01] | **0.034** | 2.39 | [0.80, 3.98] | 0.133 |
| Base Weight | 0.96 | [0.94, 0.98] | **<0.001** | 0.95 | [0.93, 0.97] | **<0.001** |
| Session |  |  |  | 0.28 | [0.26, 0.30] | **<0.001** |
| **Random Effects** | | | | | | |
| σ^2^ | 2.11 | | | 1.53 | | |
| τ_00_ | 16.68 _Patient_ID_ | | | 16.43 _Patient_ID_ | | |
| ICC | 0.89 | | | 0.91 | | |
| N | 89 _Patient_ID_ | | | 89 _Patient_ID_ | | |
| Observations | 447 | | | 447 | | |
| Marginal R^2^ / Conditional R^2^ | 0.948 / 0.994 | | | 0.951 / 0.996 | | |
| AIC | 1917.51 | | | **1801.92** | | |
| BIC | 1933.92 | | | 1822.44 | | |

*Note.* ED = Eating Disorder; CI = Confidence Interval; ICC = Intraclass Correlation Coefficient; AIC = Akaike Information Criterion; BIC = Bayesian Information Criterion.

| **Weight (of Non-Restrictive EDs) Model Comparisons** | | | | | | |
| --- | --- | --- | --- | --- | --- | --- |
|  | **Null Model** | | | **Full Model** | | |
| *Predictors* | *Estimates* | *95% CI* | *p* | *Estimates* | *95% CI* | *p* |
| (Intercept) | 2.09 | [1.42, 2.76] | **0.002** | 2.15 | [1.45, 2.85] | **0.002** |
| Base Weight | 0.98 | [0.97, 0.98] | **<0.001** | 0.98 | [0.94, 1.02] | **<0.001** |
| Session |  |  |  | -0.01 | [-0.05, 0.03] | 0.775 |
| **Random Effects** | | | | | | |
| σ^2^ | 11.54 | | | 11.54 | | |
| τ_00_ | 4.26 _Patient_ID_ | | | 4.26 _Patient_ID_ | | |
| ICC | 0.27 | | | 0.27 | | |
| N | 138 _Patient_ID_ | | | 138 _Patient_ID_ | | |
| Observations | 888 | | | 888 | | |
| Marginal R^2^ / Conditional R^2^ | 0.986 / 0.990 | | | 0.986 / 0.990 | | |
| AIC | **4856.84** | | | 4858.76 | | |
| BIC | 4876.00 | | | 4882.71 | | |

*Note.* ED = Eating Disorder; CI = Confidence Interval; ICC = Intraclass Correlation Coefficient; AIC = Akaike Information Criterion; BIC = Bayesian Information Criterion.

| **Predictors of Treatment Outcome Model Comparisons** | | | | | | | | | | | | | | | | | | |
| --- | --- | --- | --- | --- | --- | --- | --- | --- | --- | --- | --- | --- | --- | --- | --- | --- | --- | --- |
|  | **Null Model** | | | **Restrictive ED** | | | **Zone** | | | **GAD-7** | | | **GAD-7 + PHQ-9** | | | **GAD-7 + PHQ-9 + Early Change** | | |
| *Predictors* | *Estimates* | *95% CI* | *p* | *Estimates* | *95% CI* | *p* | *Estimates* | *95% CI* | *p* | *Estimates* | *95% CI* | *p* | *Estimates* | *95% CI* | *p* | *Estimates* | *95% CI* | *p* |
| (Intercept) | 0.72 | \| [0.45, 0.97] \| \| --- \| \|  \| \|  \| | **0.008** | 0.70 | [0.42, 0.98] | **0.013** | 0.65 | [0.32, 0.97] | 0.052 | 0.57 | [0.29, 0.85] | **0.044** | 0.45 | [0.17, 0.73] | 0.108 | 0.69 | [0.51, 0.86] | **<0.001** |
| Base EDE-Q | 0.67 | \| [0.67, 0.69] \| \| --- \| \|  \| | **<0.001** | 0.67 | [0.60, 0.74] | **<0.001** | 0.67 | [0.60, 0,74] | **<0.001** | 0.61 | [0.54, 0.68] | **<0.001** | 0.58 | [0.51, 0.65] | **<0.001** | 0.88 | [0.83, 0.93] | **<0.001** |
| Session | -0.14 | [-0.15, -0.13] | **<0.001** | -0.14 | [-0.15, -0.13] | **<0.001** | -0.14 | [-0.15, -0.13] | **<0.001** | -0.14 | [-0.15, -0.13] | **<0.001** | -0.14 | [-0.15, -0.13] | **<0.001** | -0.14 | [-0.15, -0.13] | **<0.001** |
| Restrictive ED [1] |  |  |  | 0.06 | [-0.11, 0.23] | 0.720 |  |  |  |  |  |  |  |  |  |  |  |  |
| Zone [1] |  |  |  |  |  |  | 0.18 | [-0.06, 0.42] | 0.435 |  |  |  |  |  |  |  |  |  |
| Zone [2] |  |  |  |  |  |  | -0.04 | [-0.37, 0.29] | 0.897 |  |  |  |  |  |  |  |  |  |
| Zone [3] |  |  |  |  |  |  | 0.00 | [-0.32, 0.32] | 0.990 |  |  |  |  |  |  |  |  |  |
| Zone [4] |  |  |  |  |  |  | -0.16 | [-0.45, 0.13] | 0.594 |  |  |  |  |  |  |  |  |  |
| Base GAD-7 |  |  |  |  |  |  |  |  |  | 0.03 | [0.01, 0.05] | 0.077 | -0.01 | [-0.03, 0.01] | 0.660 | 0.01 | [0.00, 0.02] | 0.424 |
| Base PHQ-9 |  |  |  |  |  |  |  |  |  |  |  |  | 0.05 | [0.03, 0.07] | **0.017** | 0.00 | [-0.01, 0.01] | 0.868 |
| Early Change |  |  |  |  |  |  |  |  |  |  |  |  |  |  |  | -0.88 | [-0.93, -0.83] | **<0.001** |
| **Random Effects** | | | | | | | | | | | | | | | | | | |
| σ^2^ | 0.40 | | | 0.40 | | | 0.40 | | | 0.40 | | | 0.40 | | | 0.33 | | |
| τ_00_ | 0.74 _Patient_ID_ | | | 0.74 _Patient_ID_ | | | 0.72 _Patient_ID_ | | | 0.71 _Patient_ID_ | | | 0.69 _Patient_ID_ | | | 0.15 _Patient_ID_ | | |
| ICC | 0.65 | | | 0.65 | | | 0.65 | | | 0.64 | | | 0.63 | | | 0.31 | | |
| N | 155 _Patient_ID_ | | | 155 _Patient_ID_ | | | 155 _Patient_ID_ | | | 155 _Patient_ID_ | | | 155 _Patient_ID_ | | | 155 _Patient_ID_ | | |
| Observations | 344 | | | 344 | | | 344 | | | 344 | | | 344 | | | 344 | | |
| Marginal R^2^ / Conditional R^2^ | 0.417 / 0.797 | | | 0.419 / 0.797 | | | 0.424 / 0.796 | | | 0.429 / 0.796 | | | 0.450 / 0.799 | | | 0.748 / 0.827 | | |
| AIC | 903.48 | | | 905.35 | | | 908.86 | | | 902.38 | | | 898.70 | | | **718.11** | | |
| BIC | 922.69 | | | 928.40 | | | 943.42 | | | 925.42 | | | 925.59 | | | 748.83 | | |

*Note.* EDE-Q = Eating Disorder Examination Questionnaire; ED = Eating Disorder; GAD-7 = Generalized Anxiety Scale; PHQ-9 = Patient Health Questionnaire; CI = Confidence Interval; ICC = Intraclass Correlation Coefficient; AIC = Akaike Information Criterion; BIC = Bayesian Information Criterion.

| **Predictors of Withdrawal Model Comparisons** | | | | | | | | | | | | | | | | | | |
| --- | --- | --- | --- | --- | --- | --- | --- | --- | --- | --- | --- | --- | --- | --- | --- | --- | --- | --- |
|  | **Null Model** | | | **Restrictive ED** | | | **Restrictive ED + Zone** | | | **Restrictive ED + Base EDE-Q** | | | **Restrictive ED + PHQ-9** | | | **Restrictive ED + PHQ-9 + GAD-7** | | |
| *Predictors* | *Odds Ratios* | *95% CI* | *p* | *Odds Ratios* | *95% CI* | *p* | *Odds Ratios* | *95% CI* | *p* | *Odds Ratios* | *95% CI* | *p* | *Odds Ratios* | *95% CI* | *p* | *Odds Ratios* | *95% CI* | *p* |
| (Intercept) | 1.50 | [1.15, 1.95] | **0.003** | 1.09 | [0.78, 1.51] | 0.620 | 1.27 | [0.65, 2.48] | 0.480 | 1.31 | [0.51, 3.36] | 0.575 | 0.58 | [0.26, 1.30] | 0.188 | 0.61 | [0.26, 1.41] | 0.241 |
| Restrictive ED [1] |  |  |  | 2.60 | [1.44, 4.66] | **0.001** | 2.72 | [1.51, 4.89] | **0.001** | 2.57 | [1.44, 4.66] | **0.002** | 2.69 | [1.49, 4.85] | **0.001** | 2.71 | [1.51, 4.89] | **0.001** |
| Zone [1] |  |  |  |  |  |  | 0.72 | [0.32, 1.56] | 0.398 |  |  |  |  |  |  |  |  |  |
| Zone [2] |  |  |  |  |  |  | 0.78 | [0.26, 2.33] | 0.657 |  |  |  |  |  |  |  |  |  |
| Zone [3] |  |  |  |  |  |  | 0.94 | [0.31, 2.88] | 0.917 |  |  |  |  |  |  |  |  |  |
| Zone [4] |  |  |  |  |  |  | 1.12 | [0.43, 2.92] | 0.820 |  |  |  |  |  |  |  |  |  |
| Base EDE-Q |  |  |  |  |  |  |  |  |  | 0.95 | [0.77, 1.18] | 0.679 |  |  |  |  |  |  |
| Base PHQ-9 |  |  |  |  |  |  |  |  |  |  |  |  | 1.04 | [1.00, 1.08] | 0.098 | 1.05 | [0.97, 1.14] | 0.151 |
| Base GAD-7 |  |  |  |  |  |  |  |  |  |  |  |  |  |  |  | 0.98 | [0.91, 1.06] | 0.684 |
| Observations | 230 | | | 230 | | | 230 | | | 230 | | | 230 | | | 230 | | |
| R^2^ Tjur | 0.000 | | | 0.046 | | | 0.053 | | | 0.046 | | | 0.056 | | | 0.057 | | |
| AIC | 311.58 | | | 302.75 | | | 309.08 | | | 304.58 | | | **301.96** | | | 303.80 | | |
| BIC | 315.02 | | | 309.64 | | | 329.71 | | | 314.90 | | | 312.28 | | | 317.55 | | |

*Note.* EDE-Q = Eating Disorder Examination Questionnaire; ED = Eating Disorder; GAD-7 = Generalized Anxiety Scale; PHQ-9 = Patient Health Questionnaire; CI = Confidence Interval; ICC = Intraclass Correlation Coefficient; AIC = Akaike Information Criterion; BIC = Bayesian Information Criterion.

| **Predictors of Treatment Extension Model Comparisons** | | | | | | | | | | | | | | | | | | |
| --- | --- | --- | --- | --- | --- | --- | --- | --- | --- | --- | --- | --- | --- | --- | --- | --- | --- | --- |
|  | **Null Model** | | | **Restrictive ED** | | | **Restrictive ED + Zone** | | | **Restrictive ED + Zone + Base EDE-Q** | | | **Restrictive ED + Zone + Base EDE-Q + GAD-7** | | | **Restrictive ED + Zone + Base EDEQ + PHQ-9 + GAD-7** | | |
| *Predictors* | *Odds Ratios* | *95% CI* | *p* | *Odds Ratios* | *95% CI* | *p* | *Odds Ratios* | *95% CI* | *p* | *Odds Ratios* | *95% CI* | *p* | *Odds Ratios* | *95% CI* | *p* | *Odds Ratios* | *95% CI* | *p* |
| (Intercept) | 0.13 | [0.09, 0.19] | **<0.001** | 0.17 | [0.11, 0.27] | **<0.001** | 0.07 | [0.02, 0.27] | **<0.001** | 0.01 | [0.00, 0.08] | **<0.001** | 0.01 | [0.00, 0.12] | **<0.001** | 0.01 | [0.00, 0.12] | **<0.001** |
| Restrictive ED [1] |  |  |  | 0.38 | [0.14, 1.06] | 0.063 | 0.31 | [0.11, 0.89] | **0.031** | 0.31 | [0.10, 0.92] | **0.035** | 0.33 | [0.11, 1.00] | **0.044** | 0.33 | [0.12, 1.00] | 0.050 |
| Zone [1] |  |  |  |  |  |  | 1.47 | [0.29, 7.44] | 0.643 | 1.45 | [0.28, 7.51] | 0.654 | 1.49 | [0.29, 7.74] | 0.634 | 1.48 | [0.28, 7.66] | 0.640 |
| Zone [2] |  |  |  |  |  |  | 8.69 | [1.52, 49.62] | **0.015** | 10.94 | [1.83, 64.95] | **0.009** | 11.39 | [1.87, 68.94] | **0.008** | 11.67 | [1.93, 71.04] | **0.008** |
| Zone [3] |  |  |  |  |  |  | 2.08 | [0.27, 16.25] | 0.483 | 1.75 | [0.22, 13.98] | 0.596 | 2.12 | [0.26, 17.24] | 0.484 | 2.11 | [0.26, 17.07] | 0.486 |
| Zone [4] |  |  |  |  |  |  | 6.71 | [1.31, 34.01] | **0.022** | 7.11 | [1.37, 36.83] | **0.020** | 7.27 | [1.37, 38.32] | **0.020** | 7.25 | [1.37, 38.32] | **0.020** |
| Base EDE-Q |  |  |  |  |  |  |  |  |  | 1.78 | [1.13, 2.89] | **0.017** | 2.07 | [1.22, 3.52] | **0.006** | 2.09 | [1.23, 3.56] | **0.006** |
| Base PHQ-9 |  |  |  |  |  |  |  |  |  |  |  |  | 0.94 | [0.86, 1.01] | 0.119 | 0.95 | [0.85, 1.07] | 0.378 |
| Base GAD-7 |  |  |  |  |  |  |  |  |  |  |  |  |  |  |  | 0.98 | [0.87, 1.10] | 0.723 |
| Observations | 229 | | | 229 | | | 229 | | | 229 | | | 229 | | | 229 | | |
| R^2^ Tjur | 0.000 | | | 0.016 | | | 0.089 | | | 0.116 | | | 0.129 | | | 0.130 | | |
| AIC | 164.06 | | | 162.05 | | | 155.34 | | | 150.40 | | | **149.94** | | | 151.81 | | |
| BIC | 167.50 | | | 168.92 | | | 175.94 | | | 174.43 | | | 177.41 | | | 182.71 | | |

*Note.* EDE-Q = Eating Disorder Examination Questionnaire; ED = Eating Disorder; GAD-7 = Generalized Anxiety Scale; PHQ-9 = Patient Health Questionnaire; CI = Confidence Interval; ICC = Intraclass Correlation Coefficient; AIC = Akaike Information Criterion; BIC = Bayesian Information Criterion.

**Sensitivity Analyses Model Properties of Primary and Secondary Outcome Measures across Time**

|  | *β* | 95% CI | *P* |
| --- | --- | --- | --- |
| **EDE-Q Global** | -0.12 | [-0.13, -0.11] | **<0.001** |
| **Eating Disorder Symptoms** |  |  |  |
| Binge Episodes | -0.27 | [-0.32, -0.22] | **<0.001** |
| Binge Days | -0.29 | [-0.33, -0.25] | **<0.001** |
| Vomiting Episodes | -0.38 | [-0.48, -0.28] | **<0.001** |
| Laxative Episodes | -0.007 | [-0.02, 0.007] | 0.294 |
| Driven Exercise Episodes | -0.47 | [-0.55, -0.39] | **<0.001** |
| **PHQ-9** | -0.26 | [-0.30, -0.22] | **<0.001** |
| **GAD-7** | -0.10 | [-0.14, -0.06] | **<0.001** |
| **Weight by Restrictive Categorization** |  |  |  |
| Restrictive^1^ | 0.17 | [-0.21, -0.13] | **<0.001** |
| Other^2^ | 0.04 | [-0.08, -0.00] | 0.071 |

Note:

NS: CI: Confidence Interval, EDE-Q: Eating Disorder Examination Questionnaire. PHQ-9 Patient Health Questionnaire, GAD-7 General Anxiety Disorder Questionnaire

1. Restrictive Eating Disorders characterized by significant weight loss included anorexia nervosa, atypical anorexia, and avoidant restrictive food intake disorder.
2. All other eating disorder diagnoses not included in item 1 were categorized as other.

**Marginal Means and Standard Deviations of Linear Mixed Effects Models evaluating Sensitivity Analyses**

| **Measure** | **S1** | **S4** | **S10** | **FU1** | **FU3** |
| --- | --- | --- | --- | --- | --- |
|  | ***M***  ***95% CI*** | ***M***  ***95% CI*** | ***M***  ***95% CI*** | ***M***  ***95% CI*** | ***M***  ***95% CI*** |
| **EDE-Q Global** | 3.78  [3.70, 3.86] | 2.96  [2.88, 3.05] | 2.21  [2.13, 2.30] | 2.09  [2.00, 2.18] | 1.84  [1.75, 1.94] |
| **Eating Disorder Symptoms** | | | | | |
| Binge Episodes | 12.28  [11.57, 12.99] | 4.07  [3.75, 4.39] | 2.47  [2.15, 2.79] | 2.20  [1.86, 2.54] | 1.67  [1.27, 2.07] |
| Binge Days | 11.37  [10.80, 11.94] | 3.57  [3.29, 3.84] | 1.84  [1.56, 2.11] | 1.55  [1.25, 1.84] | 0.97  [0.62, 1.32] |
| Vomiting Episodes | 10.73  [9.43, 12.03] | 5.81  [5.17, 6.44] | 3.50  [2.84, 4.16] | 3.11  [2.41, 3.82] | 2.34  [1.53, 3.16] |
| Driven Exercise Episodes | 11.56  [10.57, 12.55] | 6.32  [5.70, 6.94] | 3.52  [2.88, 4.16] | 3.05  [2.38, 3.72] | 2.12  [1.36, 2.87] |
| **ED-15** | 3.93  [3.86, 4.00] | 3.37  [3.27, 3.46] | 2.26  [2.17, 2.36] | 2.08  [1.98, 2.17] | 1.71  [1.61, 1.82] |
| **GAD-7** | 13.21  [12.88, 13.54] | 11.10  [11.00, 11.30] | 10.60  [10.40, 10.70] | 10.50  [10.30, 10.70] | 10.30  [10.00, 10.50] |
| **PHQ-9** | 15.10  [14.73, 15.47] | 13.60  [13.30, 13.90] | 12.10  [11.80, 12.40] | 11.80  [11.60, 12.10] | 11.40  [11.00, 11.70] |

Note:

EDE-Q: Eating Disorder Examination Questionnaire, GAD-7: General Anxiety Disorder Questionnaire, PHQ-9 Patient Health Questionnaire.
